# Can Intensive Use of Alcohol-Based Hand Rubs Lead to Passive Alcoholization?

**DOI:** 10.3390/ijerph7083038

**Published:** 2010-07-30

**Authors:** Vincent Bessonneau, Michel Clément, Olivier Thomas

**Affiliations:** Environment and Health Research Laboratory, French School of Public Health, Avenue du Professeur Léon Bernard, 35043 Rennes Cedex, France; E-Mails: vincent.bessonneau@ehesp.fr (V.B.); michel.clement@ehesp.fr (M.C.)

**Keywords:** alcohol-based hand rubs, passive alcoholization, exposure, health-care workers, indoor air

## Abstract

Hand disinfection with alcohols-based hand rubs (ABHRs) are known to be the most effective measure to prevent nosocomial infections in healthcare. ABHRs contain on average 70% by weight of one or more alcohols. During the hand rubbing procedure, users are exposed to these alcohols not only through dermal contact, but also via inhalation, due to the physical and chemical properties of alcohols volatilizing from alcoholic solutions or gels into the air. Ethanol ingestion is well known to increase risks of several diseases (affecting the pancreas, liver, cardiovascular system…), but there is a lack of knowledge about the effects of exposure to other alcohols (including *n*- or isopropanol) via inhalation and dermal contact, despite the worldwide use of ABHRs. This work aims at discussing possible health effects related to unintentional alcoholization (via inhalation and dermal contact) from professional ABHR usage to suggest the need for more research in this area (but not to question the value of ABHRs). Based upon an average of 30 hand rubbings per healthcare professional per day, it can be assumed that a healthcare worker may be exposed to a maximum 5,500 mg/m^3^ per work shift, five times above the recommended occupational time weighted average limit. Thus, in order to answer the question posed in the title, studies on spatial and temporal variability of alcohol emission from ABHRs in real world situations and studies on certain high risk individuals are needed.

## 1. Introduction

The effect of hand hygiene interventions on rates of gastrointestinal and respiratory illnesses is well known. Moreover, hand hygiene is the simplest and most effective measure to reduce hospital-acquired infections [1]. During patient care, the risk of hands contamination depends on the type of nursing activity. “Dirty activities” (e.g., washing incontinent patients) are higher risk than “clean activities” (e.g., taking a patient’s pulse or oral temperature). For many decades, hygienic hand washing with non-medicated or medicated soap and water were regarded in many countries as the best method to prevent nosocomial infections in healthcare [2]. Since the 1960s and the commercialization of the first alcohol-based liquid cleanser (Sterillium), alcoholic solutions are more and more used for hand disinfection [2,3]. Several *in vitro* and *in vivo* studies have indicated considerably better antimicrobial killing with the use of alcohol-based hand rubs (ABHRs) than standard hand washing with soaps [4–6]. Alcohols are bactericidal, virucidal, myobactericidal and fungicidal [7]. In addition, antiseptic soaps have other significant disadvantages compared to ABHRs, such as skin irritation [8–10], the need for access to a sink with water supply for washing and rinsing [7], or the longer time spent on the hand washing procedure [11]. In the light of these studies, the CDC has published guidelines for hand hygiene in healthcare [12] clearly favoring the use of ABHRs over antimicrobial soaps. Although the frequency of hygienic hand disinfection depends on the nature of activities and the compliance rate within each healthcare service, Voss and Widmer [11] have estimated that on average 20 hand disinfections are carried out per healthcare worker per shift.

It is well documented that chronic alcohol ingestion is correlated with an increased risk of cardiovascular, pancreas or liver diseases, and psychological disorders [13]. Damage to the central nervous system and to the peripheral one can also occur from alcohol misuse. The health effects of alcohol ingestion have led the International Agency for Research on Cancer (IARC) to classify ethanol and alcoholic beverages as Group 1 carcinogens [14].

Contrary to alcohol ingestion, there is limited data regarding inhalation and dermal exposure to alcohol. Given the health effects of alcohol ingestion, it can be assumed that alcohol absorption throughout inhalation and in a lesser extent via dermal contact might induce the same health negative effects in the long term. Kramer *et al.* [15] reported that the quantity of ethanol absorbed after excessive hand disinfection is below toxic levels for humans. In context of the H1N1 flu pandemic, or other coming infectious crisis, several interventions to improve compliance with hands disinfection products have been implemented for healthcare workers and people in hospitals and it can be assumed that before long ABHRs will be used more frequently and by more people. In this work, the possible passive alcoholization risk for healthcare workers caused by the use of ABHRs is discussed without questioning the importance of ABHRs to reduce cross-transmissions. Passive alcoholization refers to the unintentional alcohol intake via inhalation and/or dermal absorption.

## 2. Alcohol-Based Hands Rubs

The concept of using alcohol for hand antisepsis seems to have appeared in the early 19th century. In the 1890s and early 1900s, the germicidal activity of alcohol was demonstrated and it was proposed for use as a skin disinfectant [16]. The antimicrobial activity is due to alcohol’s (ethanol’s) ability to denature proteins [17]. Alcohols are effective against most vegetative Gram-positive and Gram-negative bacteria, many fungi, especially *Mycobacterium tubercolisis*, and a variety of enveloped (e.g., hepatitis B, human immunodeficiency virus and herpes simplex virus) and non-enveloped (e.g., enterovirus, adenovirus and rotavirus) viruses [18,19]. Most ABHRs contain one or more alcohols including ethanol, *n*-propanol and isopropanol. Table 1 provides physical and chemical characteristics of alcohols used in ABHR formulation [20–25].

**Figure 1.**
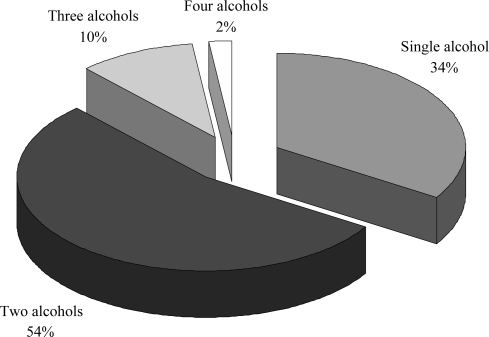
Breakdown of the different ABHR formulations. Data from SFHH [26].

Ethanol, *n*-propanol and isopropanol are the most volatile compounds, as proven by their Henry’s constant. Henry’s constant represents the solubility of a chemical compound in a liquid at a particular temperature. This constant reflects the relative volatility of a chemical compound. Some 54% of commercially available alcohol products are made up by two different alcohols (Figure 1), and ethanol and isopropanol are the most used components (Table 2).

Ethanol is used almost equally in the formulation of the four categories of ABHRs, depending on the number of alcohols (one, two, three or four). Isopropanol is mainly used in the single alcohol category. Other active ingredients, such as chlorhexidine or triclosan may be added to ensure a residual antimicrobial activity [19,26]. Besides ABHRs, alcohol-free hand hygiene products containing benzalkonium chloride or chlorhexidine have been proposed [19]. A few studies have reported that these products are less effective in preventing cross-transmission of pathogens [1,3,27]. Since the 2000s, several studies have emphasized the importance of high compliance with ABHR usage as a hand hygiene program to reduce nosocomial infections [2,28–30]. Scheithauer *et al.* [31] have observed a regular 78% increase of ABHR usage in intensive care units between 2003 and 2008. A recent review has found an overall median compliance rate with hand hygiene guidelines in hospital care of 40% [32].

## 3. Intentional Alcohol Intake

**Figure 2.**
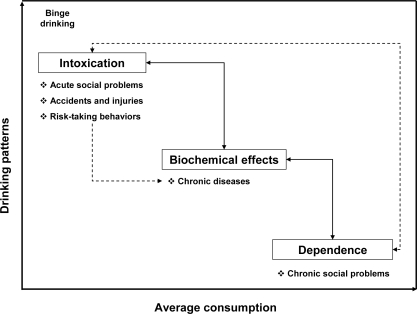
Overview of alcohol-related harmful mechanisms (adapted from Rehm *et al.* [33]).

Intentional alcohol intake defines the consumption of alcoholic beverages, used in many societies for many purposes. Alcohol consumption is related to a wide range of physical, mental and social harms. As shown in Figure 2, the link between alcohol consumption and health consequences depends on the average volume of consumption, drinking patterns, and on the mediating mechanisms: intoxication, dependence, and biochemical effects [33]. Alcohols-related harms are mediated by three mechanisms: intoxication, dependence and biochemical effects.

Intoxication is an acute disease listed in the 10th revision of the International Classification of Diseases and Related Health Problems (ICD-10), and occurring after ingestion of a large amount of alcoholic beverages in a limited period of time [34]. Most of the symptoms of alcohol intoxication are due to the effects of alcohol on the central nervous system.

Alcohol dependence has been classified in the 9th revision of the International Classification of Diseases and Related Health Problems (ICD-9) as a mental disorder. The action of alcohol on the brain induces complex changes in brain chemistry and lead to neuroadaptation, increasing alcohol tolerance [35,36].

The biochemical effects of alcohol seem to influence chronic disease in harmful ways [33]. Increased rates of heart attacks, hypertension and other cardiovascular diseases are well associated with heavy drinking episodes [37–39]. Alcohol is a potent teratogen and high consumption of alcoholic beverages during pregnancy leads to fetal alcohol syndrome (FAS), characterized by growth deficiencies, craniofacial abnormalities, prematurity and serious neurobiological dysfunctions, including mental retardation [40,41]. Repeated alcohol consumption has been estimated as the major cause of liver cirrhosis [42]. Long term alcohol misuse during adolescence impairs brain development and increases neuropsychatric and cognitive disorders [43,44]. Chronic consumption can also cause thiamine deficiency inducing neurological disorder known as Wernicke-Korsakoff Syndrome (WKS) [45,46]. WKS is a combination of Wernicke’s encephalopathy (WE) and Korsakoff’s psychosis and the main symptoms include mental confusion, oculomotor disturbances, behavioral abnormalities and memory impairments [45,47]. The International Agency for Research on Cancer (IARC) has classified alcohol drinking as carcinogenic to humans [14]. Alcohol is recognized as a risk factor of several cancers: mouth (lip and tongue), pharynx, larynx, hypopharynx, esophagus, liver, breast, stomach, pancreas, colon, rectum, prostate, salivary glands, ovarium, endometrium and bladder [14,48–53]. Finally, cancer risks appear to increase with increasing volume of alcohol consumed [50]. The main chronic diseases related to alcohol drinking are reported in Table 3.

## 4. Unintentional Alcohol Intake

Alcohols, as chemical substances, are widely used as solvents in the paint, adhesive, varnish, ink, cosmetic and perfume industry, and as disinfectants in cleaning products. Few studies have focused on occupational exposure to alcohols [54–57]. Brugnone *et al.* [54] have sampled isopropanol in air, breath, blood and urine to assess the occupational exposure of 12 workers in a print works. The authors reported an isopropanol concentration range between 7 and 645 mg/m^3^ in air samples, and between 4 and 437 mg/m^3^ in breath samples, but with no detection in urine and blood. They have also observed a significant correlation between environmental and exhaled air concentrations.

During the 1950s and 1960s; floor layers used to handle between 20 and 30 L per day of alcohol-based glues [55,56]. In the early 1970s, an exposure assessment measured ethanol or methanol levels around 500 mg/m^3^ [57]. Since the 1970s, efforts have been made to reduce exposure of floor layers to organic solvents, and alcohol-based glues have been substituted by water-based glues or solvents with low volatility and new types of glues have been designed. In addition, since the 1980s, floor layers typically wear protective masks containing charcoal filters [56].

Cumulative occupational and home exposures to well-known irritants, such as isopropyl alcohol, can cause respiratory system irritations. Tonini *et al.* [58] have reported a case of vocal cord dysfunction, diagnosed in a nurse in charge of cleaning endoscopy instruments. As consequence, reprocessing of instruments in washer disinfectors is strongly recommended.

Some healthcare workers have complained of an unpleasant smell associated with the use of alcohol-based products use like ABHRs [1]. During hand rubbing, users are exposed to different types of alcohols (e.g., ethanol, *n*-propanol and isopropanol) via inhalation and dermal contact. Depending on manufacturer’s recommendations, a good hand disinfection procedure is generally achieved with a 30 second hand rubbing with 3 mL of alcohol-based products. Some manufacturers recommend doing this procedure twice [26]. Under practical conditions, this procedure averages between 6 to 24 seconds [30].

The CDC hands hygiene guidelines have reported that an average of five hand rubs per shift to as many as 30 hand rubs per shift are carried out per health care worker [12]. However, this number varies markedly, depending on the nature of the clinical activity, the hospital setting, or the healthcare worker’s adherence with hands hygiene programs [30]. Indeed, the CDC hands hygiene guidelines has reported that adherence of healthcare workers with hygiene practices varies widely between 5% and 81%, with an overall average of 40% [12]. The SUMER survey conducted in 2003, has reported that healthcare workers are six times more exposed to alcohols (35% *versus* 7%) than other workers [59].

**Figure 3.**
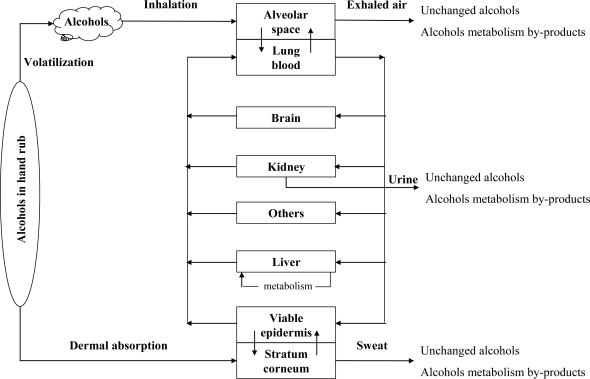
Alcohol absorption, distribution, metabolization, and excretion pathways.

ABHR users are exposed to alcohols via inhalation and dermal route. Alcohols are volatile organic oxygenated species, water soluble, and highly mobile. A schematic diagram of alcohol absorption, distribution, metabolization and excretion pathways is shown in Figure 3. Through inhalation exposure, alcohols are readily absorbed into the body via the lungs. In the alveoli, a gas-blood equilibrium is rapidly established by passive diffusion of alcohol vapors between alveolar gas and blood. To a lesser extent, alcohols are also absorbed through dermal contact, except ethanol for which percutaneous absorption is very low (about 1%) [24].

## 5. Risk Assessment

Absorbed alcohols are widely diffused throughout the organism due to their high water solubility and are rapidly distributed into highly vascular organs such as brain and liver. Alcohols are eliminated from the body mainly by metabolism. A small amount is excreted in unmetabolized form in urine, sweat and breath (2%–5%) [23,24].

Alcohols are metabolized in the liver via two different pathways: the alcohol dehydrogenase (ADH) pathway located in the cytosol of hepatocytes, and the microsomal ethanol-oxidizing system (MEOS; CYP2E1) pathway located on the endoplasmic reticulum [60]. Through both pathways ethanol, *n*-propanol and isopropanol are metabolized to acetaldehyde, propionaldehyde and acetone, respectively [22,61]. A part of the by-products formed are then eliminated from the organism via the kidneys and by exhaled air. Another part is converted to acetate and propionate by aldehyde dehydrogenase (ALDH) located in the mitochondria. The acetate and propionate produced are released into the blood and are oxidized by peripheral tissues to acetic and propionic acid and finally into carbon dioxide and water [62–65].

Alcohols have low acute toxicity by all routes of exposure. The critical effect is the irritation of respiratory system, eyes, and mucous membranes. Higher concentrations may cause central nervous system effects including dizziness, nausea, hypotension, and hypothermia. Through inhalation and dermal contact, IARC has classified isopropanol in Group 3 (inadequate evidence for carcinogenicity to humans), whereas *n*-propanol and ethanol are not evaluated as chemical substances.

On the basis of eye, nose and throat irritation, the American Conference of Governmental Industrial Hygienists (ACGIH) has recommended a threshold limit value of 1,000 ppm, 200 ppm and 400 ppm for ethanol, *n*-propanol and isopropanol, respectively, in air over an 8-hours exposure [or time-weighted average limit (TWA)], as summarized in Table 4.

For *n*-propanol and isopropanol, 15 min short-term exposure levels (STEL) of 250 ppm and 500 ppm have been added, respectively. In France, the same TWA limits for ethanol, *n*-propanol and isopropanol have been recommended, and a 15 min STEL of 5,000 ppm for ethanol has been proposed. Whereas acute and chronic health effects resulting from alcoholic beverage consumption are well known, there is a lack of knowledge regarding exposure via the inhalation and dermal routes. Despite intensive use of ABHRs in health-care, and peoples’ growing interest in these products, only a few studies have addressed the issue of alcohol intake during hand rubbing procedures [15,66]. Kramer *et al.* [15] assessed the ethanol absorption level during hand hygiene and surgical disinfection procedures. They have tested three ABHRs containing 95 % and 85 % w/w ethanol, and 55% w/w ethanol with 10% w/w *n*-propanol. The authors reported that the total amount of alcohol absorbed ranged from 358 to 1,365 mg and from 477 to 1,542 mg, respectively, after 20 hygienic and 10 surgical hand disinfections. Miller *et al.* [66] have also investigated blood ethanol concentrations before and after 50 applications of 5 mL of 62 % ethanol products in five volunteers. They have observed a blood ethanol level lower than 50 mg/L in all five participants. Both studies have concluded that ethanol absorption is below the toxic levels for humans.

These studies on blood ethanol concentrations resulting from intensive hand rub applications over a limited period of time [15–66] have in common one major limitation, the use of only ethanol-based hand rubs, whereas, as described in Section 2, most sanitizers used nowadays are made up of at least two different alcohols, typically ethanol and isopropanol, the latter producing irritation of the respiratory system and damage to the central nervous system, and being classified in Group 3 by IARC [62–64].

Finally, a simple theoretical mass balance calculation of isopropanol during hand rubbing can be considered, as proposed by Kramer *et al.* [15] (this could be extended to other alcohols). If for example a health-care worker applies 90 mL (3 mL × 30 daily hand rubs) of a 70% w/w isopropanol hand rub per shift, a maximum of 67 g will evaporate into the air. If no air exchange takes place in a 12 m^3^ room, a maximal isopropanol concentration of 5,500 mg/m^3^ in air will result, which is approximately five times above the recommended occupational TWA (980 mg/m^3^). This calculation is the worst case based on lack of air movement. Nowadays, hospital facilities have air movement from heaters and air conditioners blowing air. However, this result shows that there is a need to characterize indoor air contamination close to users, assessing spatial and temporal variability of alcohols in air. Evaporation of alcohols during hand disinfection is a localized discontinuous source of pollution and may lead to a continuous and diffuse background contamination in intensive rubbing rooms, so ABHR users might be exposed during hand rubbing to passive alcoholization.

## 6. Conclusions

Ingestion of alcohol (ethanol) is well known to cause adverse health effects such as liver cirrhosis, fetal alcohol syndrome and cancer, but there is no evidence to suggest intoxication or dependence could occur with use of ABHRs. The only issue of passive alcoholization would relate to its biochemical effects. In addition, the use of ABHRs in healthcare settings as part of a hand hygiene program has a definable, clear-cut value, while the questions being raised in this article are preliminary and the answers are far from being settled.

In a context of an increased use of ABHRs, the issue of exposure to alcohols mainly via inhalation but also through dermal absorption should be considered to determine how safe air is. Despite the existence of a few studies, there is a general lack of knowledge about alcohol, especially *n*-propanol and isopropanol, contamination levels in the environment of ABHR users such as health care workers. Thus, more research is needed for contamination assessment, including spatial and temporal variability of alcohol emissions from ABHRs to indoor air (peak *vs* average concentrations) in real world situations. In addition, the sampling and analysis of alcohols and related metabolized by-products in exhaled air of non-drinkers might be used as an exposure biomarker, as a complement to serum alcohol levels. The next layer of studies could be performed on individuals with known liver disease to see if their ability to detoxify minute amounts of alcohol would put that at special risk. These data could improve our knowledge about exposure to alcohols through the inhalation route linked to the frequent use of ABHRs, in order to be able to propose recommendations such as increases in the air exchange rate within healthcare settings, if needed.

## Figures and Tables

**Table 1.** Physical and chemical properties of alcohols used in ABHR formulation.

**Compounds**	**Molecular weight (g/mol)**	**Structural formula**	**Water solubility at 25°C (mg/L)**	**Henry’s constant at 25°C (atm.m^3^/mol)**
Ethanol	46.07	CH_3_-CH_2_OH	Fully miscible	5 × 10^−6^
*n*-Propanol	60.1	CH_3_-CH_2_-CH_2_OH	Fully miscible	7.41 × 10^−6^
Isopropanol	60.1	CH_3_-CH_2_OH-CH_3_	Fully miscible	8.10 × 10^−6^
Aminomethylpropanol	89.14	CH_3_-C(CH_3_)(NH_2_)-CH_2_OH	Fully miscible	6.48 × 10^−10^
Benzylalcohol	108.14	Ph-CH_2_OH	42.9	3.37 × 10^−7^
Phenoxyethanol	138.17	Ph-O-CH_2_-CH_2_OH	26,700	4.72 × 10^−8^

**Table 2.** Distribution of alcohols, in percentage (%), among different formulations: single (1), two (2), three (3), and four (4) alcohol-based hand rubs. Data from SFHH [26].

**Compounds**	**1**	**2**	**3**	**4**	**Total**
Ethanol	25%	46%	29%	25%	39%
n-Propanol	6%	9%	0%	25%	8%
Isopropanol	71%	39%	21%	25%	40%
Aminomethylpropanol	0%	0%	14%	0%	2%
Benzyl alcohol	0%	0%	7%	0%	1%
Phenoxyethanol	0%	6%	29%	25%	9%

**Table 3.** Summary of the main chronic diseases link to alcohol consumption.

**Main chronic diseases**	**References (selection)**
Liver cirrhosis	[42]
Fetal Alcohol Syndrome (FAS)	[40,41]
Cancer	[14,48–53]
Cardiovascular disorders	[37–39]
Neurological disorders	[43–47]

**Table 4.** Recommended alcohol occupational exposure limit values.

**Compounds**	**Country**	**8-hour time-weighted average (TWA)**		**15 min short-term exposure level (STEL)**	

		**ppm**	**mg/m^3^**	**ppm**	**mg/m^3^**
Ethanol	France	1,000	1,950	5,000	9,500
	United States	1,000	1,950	ND	ND
*n*-Propanol	France	200	500	ND	ND
	United States	200	500	250	625
Isopropanol	France	400	980	ND	ND
	United States	400	980	500	1,225

ND: no data; TWA: time-weighted average; STEL: short-term exposure limit.
